# Which ‘imperfect vaccines’ encourage the evolution of higher virulence?

**DOI:** 10.1093/emph/eoac015

**Published:** 2022-04-26

**Authors:** James J Bull, Rustom Antia

**Affiliations:** 1 Department of Biological Sciences, University of Idaho, Moscow, ID 83844-3051, USA; 2 Department of Biology, Emory University, Atlanta, GA 30322, USA

**Keywords:** Marek’s disease virus, virus evolution, prediction, mathematical models, immunity

## Abstract

**Background and objectives:**

Theory suggests that some types of vaccines against infectious pathogens may lead to the evolution of variants that cause increased harm, particularly when they infect unvaccinated individuals. This theory was supported by the observation that the use of an imperfect vaccine to control Marek’s disease virus in chickens resulted in the virus evolving to be more lethal to unvaccinated birds. This raises the concern that the use of some other vaccines may lead to similar pernicious outcomes. We examine that theory with a focus on considering the regimes in which such outcomes are expected.

**Methodology:**

We evaluate the plausibility of assumptions in the original theory. The previous theory rested heavily on a particular form of transmission–mortality–recovery trade-off and invoked other assumptions about the pathways of evolution. We review alternatives to mortality in limiting transmission and consider evolutionary pathways that were omitted in the original theory.

**Results:**

The regime where the pernicious evolutionary outcome occurs is narrowed by our analysis but remains possible in various scenarios. We propose a more nuanced consideration of alternative models for the within-host dynamics of infections and for factors that limit virulence. Our analysis suggests imperfect vaccines against many pathogens will not lead to the evolution of pathogens with increased virulence in unvaccinated individuals.

**Conclusions and implications:**

Evolution of greater pathogen mortality driven by vaccination remains difficult to predict, but the scope for such outcomes appears limited. Incorporation of mechanistic details into the framework, especially regarding immunity, may be requisite for prediction accuracy.

**Lay Summary:**

A virus of chickens appears to have evolved high mortality in response to a vaccine that merely prevented disease symptoms. Theory has predicted this type of evolution in response to a variety of vaccines and other interventions such as drug treatment. Under what circumstances is this pernicious result likely to occur? Analysis of the theory in light of recent changes in our understanding of viral biology raises doubts that medicine-driven, pernicious evolution is likely to be common. But we are far from a mechanistic understanding of the interaction between pathogen and host that can predict when vaccines and other medical interventions will lead to the unwanted evolution of more virulent pathogens. So, while the regime where a pernicious result obtains may be limited, caution remains warranted in designing many types of interventions.

## INTRODUCTION

A somewhat recent and unexpected discovery, one with potentially profound public health ramifications, is that the vaccine given to defend against symptoms of Marek’s disease virus (MDV), administered on a global scale to billions of chickens in the poultry industry, has resulted in the evolution of a highly virulent wild-type virus [1]. Not only did the evolved virus evade vaccine-immunity and cause disease in vaccinated birds, but it also killed unvaccinated birds far faster and with more certainty than did the original strain [1]. If this evolutionary process were to repeat itself for any widely-used human vaccine, it could ultimately limit the efficacy of the vaccine, ‘addict’ civilization to the vaccine, potentially causing severe disease in unvaccinated individuals.

At face value, the evidence from almost a century of human vaccines and of viruses from their pre-vaccine eras is that such a pernicious outcome has not happened or at least has not been reported—vaccines developed half a century ago continue to provide protective immunity. Whether vaccines that were effective initially continue to be effective, disease symptoms for the non-vaccinated have not gotten much worse [2–5]. Furthermore, this absence of (or weak) virulence evolution appears to apply even for imperfect vaccines (such as the influenza vaccine and pneumonia vaccine) that do not provide lifelong sterilizing immunity. It is of course difficult to detect subtle changes in virulence amid strain evolution and changes in host immunity, but the response in both cases appears to be changes in strain serotype rather than increases in virulence. With pneumonia, at least, the strain evolution is thought to be in response to the vaccine [2, 5], whereas influenza evolution may instead be to naturally-acquired immunity [6, 7]. The pertussis vaccine is a possible exception, with evidence of somewhat higher virulence evolving in response to a vaccine [8–10]. Even more reassuring, the time scale of evolution with MDV suggests that the evolution can happen in a decade or two, and it is likely that such evolution would have manifested if it was going to happen, as several human vaccines have been around for almost a century. This combined evidence certainly ameliorates concern about a universal viral evolution ‘backlash’ in response to medical interventions, but it is not conclusive in excluding the possibility. Given the changing medical landscape and social environment affecting health, and the changing immune profiles of the population, it is usually impossible to exclude small changes in virulence, but it seems a safe conclusion that we have not experienced major increases in the virulence of pathogens in response to vaccination.

The MDV case may indeed be an exception, but it is serious enough to justify scrutiny, to understand the factors affecting whether future vaccines might lead to the evolution of higher levels of virulence in viruses. And the considerations relevant to MDV potentially also apply not only to vaccines but also interventions including drugs that target the growth of viruses in infected individuals. The potential scope of this problem is therefore large.

Our goal is to assess the regimes or conditions under which vaccines and other interventions might select for the evolution of increased pathogen-virulence—i.e. MDV-like outcomes in response to the intervention. Predicting evolution ultimately relies on models, and a mathematical model for predicting MDV outcomes was proposed two decades ago [11]. This model predicted a potentially broad range of conditions in which vaccine-induced immunity would select for an increase in virulence of these infections. Our paper revisits the Gandon *et al*. [11] model (which we henceforth designate GMNR, after the authors’ initials) to understand when it is expected to operate, thereby shedding light on whether and how the MDV case is exceptional. To anticipate our conclusion, we suggest that some kinds of interventions will indeed be prone to drive ‘pernicious’ evolution of higher virulence, but there is no reason to expect that such an evolutionary outcome is the default when using typical human vaccines. Our overall message coincides with recent posts by one of the authors of GMNR and lead author of the Marek’s study [12]; we provide a more detailed evaluation of assumptions of earlier models [11, 13, 14] and explain why they may be valid only in a restricted regime. Our intent is to expand on this earlier work and to broaden the framework for understanding virulence evolution in response to vaccines and other interventions. For convenience, our emphasis is on vaccination against viruses, but the points often apply to other types of pathogens such as bacteria and protozoa, and to other interventions such as antimicrobial treatment.

## GMNR IS A MODEL OF EVOLUTION ON TRADE-OFFS

The GMNR model is one of evolutionary optima. It assumes that a virus evolves to maximize its overall transmission—maximize its descendants. In most circumstances, this process is similar to a virus evolving to maximize its reproductive number (R_0_), which equals the number of new infections arising from an infected individual in a wholly susceptible population [15]. This is nothing more than the usual natural selection, but in this case, it is natural selection among viruses to maximize their transmission in a population of hosts. An optimum is a kind of endpoint of evolution, at which no further evolution occurs because changes in any direction leave fewer descendants. An evolutionary dynamics approach to analyze the same problem has been provided as well [3]; dynamics considerations inform whether and how fast an optimum is likely to be attained. Key to evolution in these models is that the virus is confined to a constraint function, known as a trade-off. We all understand trade-offs: driving fast gets us to our destination quickly but may injure us; driving slowly is safe but delays our arrival. The trade-off function determines what characteristics the virus can attain; the evolutionary dynamics (or optima) determine which of those characteristics will prevail through evolution.

**Figure 1.**
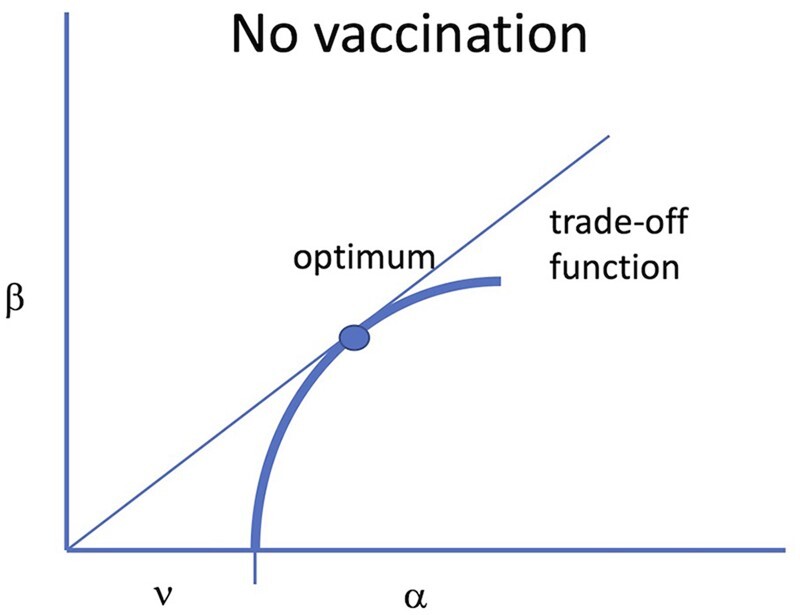
Trade-off model in which transmission rate imposes host mortality. The vertical axis is transmission rate (β) and the horizontal axis is the rate at which the infection is lost, due to a combination of host death (α) and recovery (ν). A line from the origin to any point in the space has slope β/(ν + α) and thus is proportional to the basic reproductive number (R_0_) of a virus with those parameter values. The blue curve represents a possible trade-off that the biology might impose on a virus, such that the virus cannot attain any value outside of the curve (to the upper left). The optimum is the highest value of R_0_ that the virus might attain, represented by the point at which a line through the origin is tangent to the trade-off and shown as a solid dot. In the models here and in GMNR, recovery is assumed to be independent of transmission, so the trade-off curve is bounded by ν on the horizontal axis because changes in β are assumed to affect only mortality, α. This illustration accrues to the absence of a vaccine

One way of describing the trade-offs involved with maximizing the reproductive number R_0_ is shown in Fig. 1. Using standard SIR models from epidemiology, a bit of algebra shows that R_0_ is proportional to the product of the rate of transmission per unit time which we call transmissibility (β) and the duration of infection [15, 16]. The duration of infection is determined by the rates of recovery (ν) and rate of host death caused by infection (α), and equals 1/(ν+α). R_0_ is thus proportional to β/(ν + α) in the simplest of models. There is however a trade-off assumed between β and the duration of infection. The argument goes that, for the parasite to increase transmissibility β, it must grow to higher densities in the host, but the higher density also causes an increase in α and consequently a decrease in the duration of infection [e.g. [17]]. α, the mortality rate of the infection, is commonly equated with virulence, and we likewise adopt that usage here.

GMNR considered a trade-off between mortality and transmission rate and also, briefly, a trade-off between mortality and recovery. Both trade-offs pit virulence against total transmission, whether transmission is affected directly by transmission rate or indirectly via recovery. The thick blue curve in Fig. 1 illustrates a possible trade-off between β and (ν+α). R_0_ for any given point on the curve is proportional to the slope of the line from the origin to that point, and the maximum value of R_0_ (the optimum) occurs at the point at which this line is tangent to the trade-off curve, as shown. Following most of the derivations in GMNR, the trade-off curve is bounded by ν on the horizontal axis, because it was assumed that changes in β affect α (mortality) but not recovery (ν). The same qualitative results were found by GMNR for their trade-off between recovery and virulence.

For many types of trade-off functions, there is an intermediate optimum (Fig. 1). We do not actually care about this optimum on its own. Its relevance is for comparison to what happens with an intervention—how the trade-off curve and the optimum shift. Below, we present arguments to explain when and why MDV-like outcomes are expected to evolve under the GMNR model. Our presentation is intended to appeal to intuition, and it is best considered that our graphical approach captures the spirit of their model but is not necessarily exact or as complete as theirs.

In keeping with GMNR, our arguments rest on a comparison of optima. Before embarking on this comparison, we not only acknowledge but stress that optimality may not be appropriate. First, optimality assumes knowledge of the appropriate constraint function (a trade-off), and the constraint function is usually unknown but assumed to involve host mortality. As we describe later, host mortality may not be relevant if transmission is limited, for example, by reduced host mobility associated with non-lethal infections. Second, optimality assumes that, through evolution, the virus controls its phenotype (subject to the constraints). But biophysical and biochemical constraints may limit the growth of the pathogen (bacteria do not evolve to grow infinitely fast even in rich media), and furthermore, the host may be able to block many dimensions of viral evolution. Third, the attainment of optimality takes time and may never be attained [14, 18, 19]. The limitations of optimality arguments only occasionally enter our arguments below, but the reader should be aware that those limitations offer additional reasons to doubt the generality of any evolution of virulence arguments based solely on optimality. Miller and Metcalf [10] have provided a useful analysis of outcomes when the trade-offs are ‘unbalanced’.

## MDV-LIKE OUTCOMES UNDER GMNR

**Figure 2.**
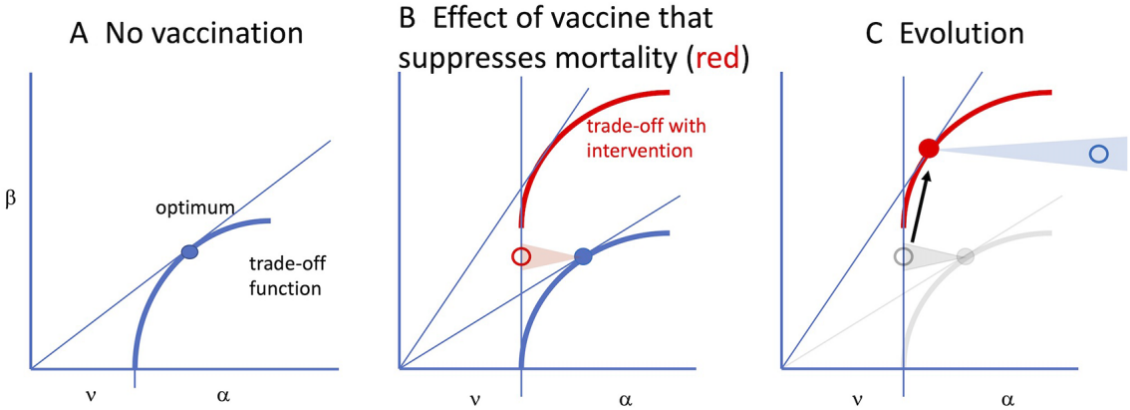
The MDV vaccine effect on evolution. ν is the recovery rate from infection, α the virus-induced death rate (virulence). Blue depicts the trade-off and optimum in the absence of vaccine, red in the presence of the vaccine. (**A**) The panel merely recapitulates Fig. 1. (**B**) With a universally-applied Marek’s-type vaccine, the trade-off shifts up because virus-induced mortality is much lower per β. Transmission rate of a virus (β) is unaffected by whether the host is vaccinated or not, but the effect of vaccination is to reduce the host death rate associated with a specific transmission rate (open red circle). (**C**) Because of the shifted trade-off, the vaccine optimum under vaccination is shifted upward, to higher β and similar or slightly lower mortality than with the original optimum. But for unvaccinated hosts, the viral optimum in red has a much higher mortality (open blue). In essence, transmission rate (tied to viral growth rate) is not affected by the vaccine, but host death is affected

We now consider different evolutionary trajectories that correspond to different scenarios for the way immunity and other interventions affect the dynamics of infection and lead to a MDV-like outcome (Fig. 2). Not all interventions are predicted by GMNR to lead to such an outcome. The GMNR theory suggests that vaccines that target the replication of the pathogen within individuals as well as toxins generated by the pathogen (their r_2_ and r_4_ effects) will lead to the evolution of pathogens with increased virulence in uninfected individuals. Our main point will be that many interventions predicted by their model to result in MDV-like evolution have a much more limited basis than first appears.

The location and shape of the trade-off function, and the evolutionary optimum, arise from the interaction between the pathogen and host. Figure 2 provides a simple illustration. To explore the effect of vaccination and other interventions on pathogen evolution—the topic considered by the GMNR model—we need to consider (i) the trade-off functions in vaccinated and unvaccinated hosts (shown by red and blue lines, respectively), (ii) the optima for the trade-offs in vaccinated and unvaccinated hosts (shown by filled circles) and (iii) how a parasite at the optimum on one trade-off behaves in the other kind of host (shown by open circles). Finally, the shaded red and blue regions and open circles represent the region of parameter space (β and α) that correspond to the outcome following infection of a treated host with a pathogen adapted to non-treated hosts (red) and vice versa (blue); thus, the open circle represents an evolved virus in a mis-matched host, and the color is that of its new environment. We note that the shaded areas indicate the directions of changes in these quantities, and the changes in the *x*-direction indicate changes in α alone and not changes in the rate of recovery.

### Interventions that reduce symptoms but not transmission

The evolutionary pathway in this example depends on an unusual type of intervention, such as that provided by the MDV vaccine. The original MDV vaccine was not the kind of vaccine to which we are accustomed with measles, flu or mumps. Its chief effect was to suppress symptoms, so that the infected chicken did not die but some transmission continued (newer MDV vaccines apparently block transmission). For there to be any appreciable evolution, the vaccine must be administered widely, to most of the population. With widespread vaccination, it is as though we moved the trade-off function vertically (Fig. 2B). For any virus, vaccination of the host means that the mortality rate imposed by a given transmission rate has just been profoundly relaxed. A virus sitting on the original optimum (solid blue) can evolve a higher transmission rate without incurring high death because the mortality associated with transmission (β) has been relaxed. Provided that the vaccine is given to most of the population, there is now selection for higher and higher transmission (with concomitant higher viral titers in the host) until symptoms of infection and transmission once again manifest themselves as host death. Evolution may return the virus to approximately the same host death rate as before—this time in vaccinated hosts (red dot in Fig. 2C)—but the virus is now transmitting faster than before.

The consequences of this evolution for the unvaccinated are clear and were identified by GMNR. The evolved virus (filled red dot) grows at much higher levels in all hosts, as given by its higher β. The death rate in the vaccinated host returns to about the same level as before (filled red). But for a host not vaccinated, the high growth of the vaccine is manifest as a vastly higher death rate—wherever the horizontal projection of the red dot would intersect the blue trade-off function (open blue). This is a simplified view of one of the ‘imperfect vaccine’ arguments of GMNR, and it depends on the vaccine being imperfect in a very specific way (an increase of r4 in their model). We next consider a different kind of imperfection that can have a similar outcome.

### Interventions that reduce growth

**Figure 3.**
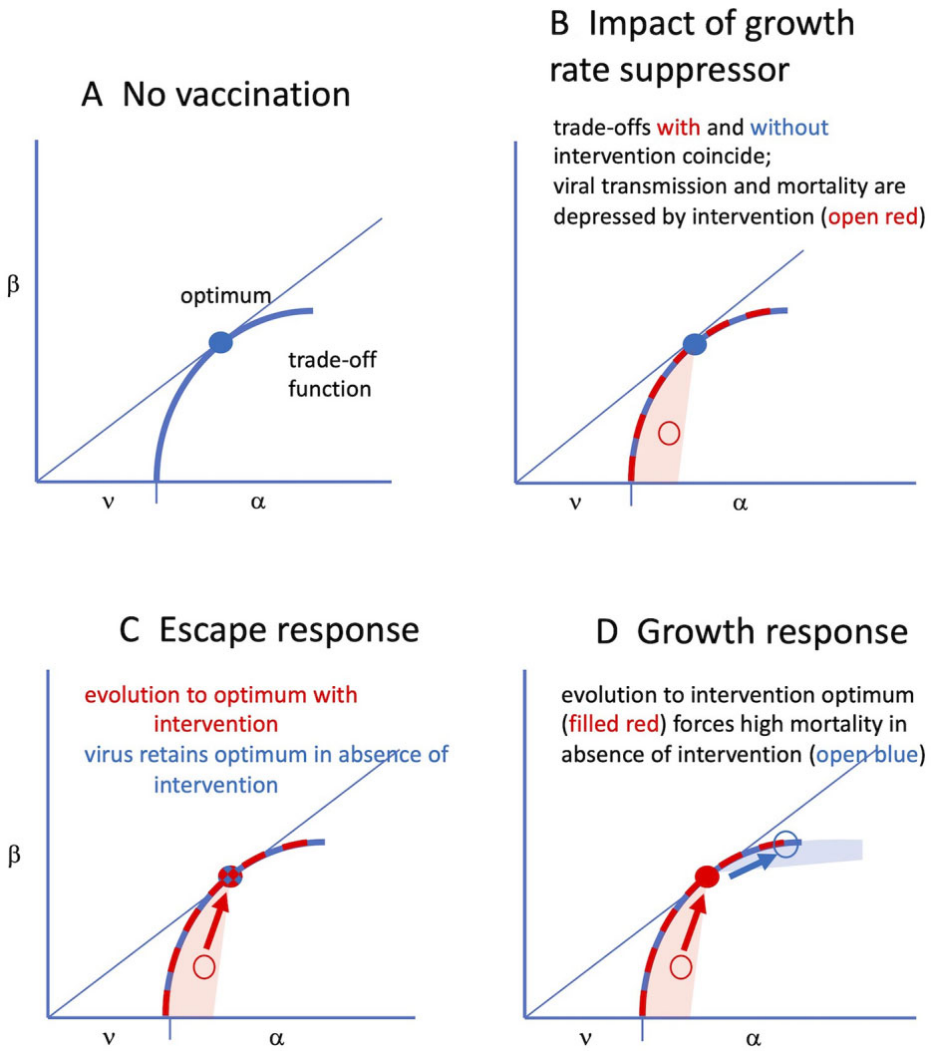
Effect of and response to an intervention that reduces viral growth rate. (**A**) Initially, the virus has evolved to its optimum in the absence of interventions (filled blue circle, as in Fig. 1). (**B**) The inhibitor suppresses viral growth rate, thereby reducing virulence (α) and reducing transmission rate (β). The performance of the inhibited virus will necessarily be suppressed to lie in a zone below and to the left of the optimum, given by open red. The trade-off itself is unaffected by the inhibitor—only the virus is affected, hence the red and blue trade-offs coincide. (**C**) The consequences of evolution to escape the inhibitor are interpreted as a virus that is no longer affected by the inhibitor. Coordinates of the virus optimum in the absence of the inhibitor coincide with those of the evolved virus in the presence of the inhibitor. Evolution to an optimum in the presence of the inhibitor is simply a return to the coordinates of the former optimum (the filled red and blue dot). (**D**) The alternative pathway of evolving a higher growth rate in response to the inhibitor leads to a new optimum in the presence of the inhibitor (filled red circle), but the evolved growth rate is higher than optimal when the inhibitor is absent (open blue). Evolution of a higher growth rate is thus potentially very harmful to hosts lacking the inhibitor. Although this figure depicts escape and growth responses as alternatives, the viral response could be a combination of escape and growth

Another way in which interventions with a MDV-like outcome might work is if the intervention reduces the rate of growth or replication of the virus in the host. Antibiotics can be considered to operate this way against bacteria. As another example, typical vaccines elicit an immunity that limits the extent of viral growth before clearance. Using our trade-off illustration, we can think of a drug or vaccine as driving the virus down the trade-off curve (Fig. 3B, open red circle): because viral growth is suppressed in the host, both the host death rate and transmission rate are suppressed. The trade-off curve itself is not necessarily affected—because the same death rate per β still operates at the host level. It is only the virus being affected, as if we have an attenuated vaccine with reduced growth and pathogenicity, but the cause is suppression by immunity or drug.

With the suppression caused by the intervention, the virus adapted to the absence of the intervention is no longer at its optimum (hence the use of an open circle in Fig. 3). Growth of the unevolved virus will be attenuated in immune or treated hosts, and this will lead to lower transmissibility as well as to lower virulence, as shown (Fig. 3B). There are at least two ways to evolve a return to the optimum. (i) Evolution via an *escape response* is to evolve so that the molecular effects of the treatment (immunity or drug) are no longer suppressive; the virus merely grows as if the drug/immunity has lost its effect (Fig. 3C). The GMNR paper specifically excluded an escape response. (ii) A *growth response*, to evolve a higher growth rate to compensate for the suppressive effect of the intervention (Fig. 3D). At the optimum, the compensated growth rate of the evolved virus in vaccinated hosts is the same as the growth rate of wild-type virus in unvaccinated individuals. Both pathways have analogy with a fish maintaining its position in a river: if the current is increased, the fish must swim faster to keep pace (growth response) or move to a site protected from the current (escape response).

The two evolutionary responses have very different consequences for hosts lacking the treatment (Fig. 3). If evolution proceeded by a growth response, the evolved virus now grows fast—faster than is optimal in the absence of the intervention (Fig. 3D), resulting in a higher mortality for the unprotected. A growth response was the one assumed by GMNR. If evolution proceeded by the escape response (with no pleiotropy—‘perfect’ escape), then both wild-type and evolved virus will have the same effect and growth rate in unvaccinated individuals, but the evolved virus will have an advantage in vaccinated individuals. Similar outcomes may be obtained for drug resistance. The virus can escape by growing faster or by changing its target so the drug no longer works. The former corresponds to the growth response and the latter to the escape response. There is no pernicious outcome for the escape response, only for the growth response (Fig. 3C versus D).

The pathway need not be either-or. A virus evolving partial escape will also be selected for faster growth to overcome immunity. The pathway followed will depend on details, but an outcome of both pathways is compatible with this theory.

### Natural immunity versus vaccines

As it applies to immunity, the theory is not specific to imperfect immunity elicited by a vaccine but should also apply to imperfect immunity from natural infection. While systemic virus infections such as measles typically give rise to long-term sterilizing immunity, many respiratory infections such as influenza or coronaviruses do not, and individuals are infected repeatedly over the course of a lifetime [20]. (We exclude SARS-CoV-1 and SARS-Cov-2 here, as they are too newly introduced to humans to have demonstrably reached an evolutionary equilibrium.) In accord with the GMNR model, the imperfect level of immunity elicited by these viruses might be expected to result in their evolving to high levels of virulence.

Broad definitive statements about the evolution of virulence of viruses causing respiratory infections (e.g. endemic human coronaviruses, influenza and human rhinoviruses) may not be within reach, but some inference is possible. People are typically infected with these viruses many times over their lifetimes [e.g. 21] and these infections for the most part exhibit relatively limited mortality. Furthermore, the emergence of new strains of these viruses (e.g. pandemic H1N1 influenza in 1918 and H3N2 influenza in 1968, SARS-CoV-1 and CoV-2 in recent decades and potentially the OC43 coronavirus in the late 1800s) resulted in severe disease during the initial pandemic, and the severity of infections has subsequently waned over time. This is the converse of the prediction of the GMNR model, which suggests that partial immunity generated by prior infections would select for higher levels of virulence. The decrease in virulence of these viruses could arise for a number of reasons. A first possible cause stems from the age-dependent severity of infections. Primary infections which typically occur in childhood are relatively mild [22, 23] and potentially elicit immunity that reduces the severity of infections when adults are re-infected [24]. Second, the viruses might have evolved to become milder, and even if current strains were to be responsible for primary infections of older individuals, these infections would be mild. Third, reinfections could be associated with antigenic escape and thus, as mentioned at the end of section ‘Interventions that reduce growth’, there would be little selection for virulence. However, an infection of finches is reported to fit the expected GMNR pattern, with imperfect immunity favoring the evolution of a more virulent pathogen [25]. This example highlights an important point relevant to medical interventions: an intervention that experiences evolution of higher virulence in the target pathogen may not be the driving factor in that evolution (i.e. the evolution could occur in the absence of the intervention).

An interesting case is provided by the ongoing evolution of myxoma virus in Australian rabbits. When first introduced in the early 1950s, the virus was highly lethal per infection (high virulence). The virulence attenuated quickly although not to low levels, and rabbit resistance evolved as well. A recent study found that myxoma virus has evolved to overcome the resistance and causes a new and highly lethal form of immune collapse [26]. These observations reinforce the message that high virulence can evolve in the absence of interventions and they further highlight the need to develop a predictive theory of virulence evolution.

## GMNR ASSUMPTIONS OF POSSIBLE LIMITED GENERALITY

Does the apparent theoretical generality of the GMNR model endow it with a potential biological robustness, and is it thus applicable to many disease agents and different types of interventions? The GMNR model specifically predicts MDV-like outcomes for only some types of interventions. As described above, the GMNR model predicts two scenarios in which an intervention will result in the evolution of the pathogen to cause higher virulence in the absence of that intervention: (i) the intervention reduces symptoms and pathology without fully reducing transmission, (ii) the intervention compromises the growth of the pathogen within the individual. As with application of any such model, the main issues are how well the disease agent matches model assumptions and whether the violated assumptions actually matter (since all assumptions are violated at some level). The challenge in assessing the model’s overall utility is that disease agents differ greatly in their biology. As such, there is no universal vulnerability of the model, because a model weakness for, say, influenza may not be a weakness for measles or malaria. Only by considering a breadth of individual cases can the model’s robustness be evaluated. We identify a few key assumptions that we suggest are not obviously general.

### Mortality limits higher transmission

The GMNR model specifically requires the penalty for increased transmission to be increased host death (higher virulence), that a higher transmission rate would incur too many excess host deaths for the gain in transmission rate (see below for a consideration of a trade-off with recovery rate). This mortality assumption has been commonplace in modeling evolution of virulence [15, 17, 27–30]. It persists as a general assumption despite limited evidence [e.g. [31]] because there has been little direct evidence to the contrary, it seems plausible and it gives substance to the model predictions (change in deaths has tangible meaning). It is perhaps surprising that, for the vast majority of human viruses, we know almost nothing about what would limit higher transmission. We do know that host death and recovery halt transmission (for most infections). But the key question for evolution of virulence is what would reduce the number of net transmissions from a virus with a higher transmission rate. The fact that dead and recovered hosts do not transmit does not tell us what offsets the gain from a higher transmission rate—except to tell us that any increases in host death or recovery will work against net transmissions.

It may seem that the only way to cast doubt on this assumption is to ascertain whether excess host death is the principal limitation to transmission of the pathogen. Here, we start by offering indirect evidence, that many human infections have too low a death rate to satisfy the GMNR model. When the case mortality from the infection is very low, say 0.001—as is approximately true for countless human respiratory and gastro-intestinal infections [at least in well-nourished populations, [4]]—we can offer a plausibility argument that mortality is not limiting higher transmission [e.g. [32]]. With a case mortality rate of 0.001, even a 10-fold increase in death rate would not be enough to offset the most modest increases in transmission rate, in which case a higher transmission rate should evolve. Furthermore, when one considers heterogeneity in the human population, bottlenecks at transmission, genetic constraints and changing host immune profiles over time, we suggest it implausible that natural selection can fine-tune an optimum to such a level. Evolutionary dynamics models likewise question the ability of fine-tuning an optimum [3, 18], as do some direct experiments with bacteriophages to test optimality [33, 34] and as does the failure of long-term adaptations of *Escherichiacoli* to reach apparent fitness maxima [35]. Furthermore, if mortality was indeed the main limit on the evolution of higher transmission for infections of minor mortality, then we should observe periodic outbreaks and pockets of virus with substantially higher death rates, much as seen for *Feline calicivirus* [36, 37]. (It is also worth noting that a high mortality rate for a virus does not imply that mortality limits the evolution of higher transmission; if mortality occurs after transmission, it is of no consequence to viral adaptation.)

GMNR alternatively analyze the effect of a trade-off between recovery rate and virulence (between our α and ν): higher virulence results in slower recovery. Recovery in the model is necessarily the rate at which the host stops transmitting, not necessarily the rate at which symptoms subside. The results are qualitatively the same with both trade-offs.

The last 1–2 decades has yielded studies formally testing trade-off models for human infections. Some have supported a mortality-transmission trade-off [38], and some have suggested alternatives, as considered next. These types of studies point a new direction in evolution of virulence work, one highly relevant to the application of GMNR. But considerable work remains.

#### Influenza and morbidity

Like many viral respiratory infections of humans, the case mortality rate of seasonal influenza is too low to suggest that host mortality is a major limit on transmission. Using self-evaluations from influenza patients admitted to hospitals, McKay *et al*. [39] found that higher infectiousness was associated with greater morbidity and with reduced patient activity. They suggested that a trade-off may exist between morbidity and transmission along the following lines: when the virus subjects the patient to increasingly severe symptoms, the patient stays home and encounters fewer contacts for transmission. Evolution along such a trade-off would prevent the virus from evolving high mortality/virulence because symptoms associated with sufficient morbidity to stay home never become extreme enough to be life threatening. It is easy to imagine that this type of trade-off could apply to many respiratory infections. There is substantial work to be done in confirming that this trade-off makes sense when considering viral titers, immune responses and measurements of morbidity by third parties, but the study at least takes the enterprise of trade-off assessments out of the realm of pure speculation and away from a focus on host death.

#### Dengue and immunity

Using serial measurements of viral titer from dengue patients, Ben-Shachar and Koelle [40] found that higher viral peaks were followed by faster declines in viral concentration. They tentatively proposed that such a pattern may reflect a trade-off between virus growth (and thus transmission rate, which is via mosquitoes) and control of the virus by immunity. That is, high viral loads, which would be associated with high virulence/death, would experience reduced transmission because of faster clearance. Note that this pattern is in the opposite direction of the trade-off between virulence and recovery assumed by GMNR. The shape and set point of this pattern would determine the optimal virulence, which could be low or high. Again, work remains in confirming the immunity-transmission trade-off, but the study again moves toward a science of formally assessing trade-offs that could be applied in models of virulence evolution.

Minimally, these different mechanisms highlight the sensitivity of GMNR predictions to limits on viral transmission. Equally, they alert us to the possibility that many viruses may not satisfy the requirement that mortality be limiting.

### Viruses are not allowed to ‘escape’ the effect of growth inhibitors

Many antimicrobial drugs suppress microbial growth, in some cases killing the microbe. Most vaccines elicit an immunity that then blocks establishment of an infection or limits its extent and duration. These interventions might be thought of as inhibitors of viral growth, and indeed, GMNR interpreted immunity that way. To be ‘imperfect’, these interventions must sometimes allow partial growth. In fact, many of them do: drugs invariably decay within the host to concentrations that allow growth; waning/incomplete immunity occurs for some vaccines and allows viral infection and replication. Both cases are encompassed in GMNR (their r2). Above, we noted two possible avenues of evolution to overcome imperfect growth suppression. One is to ‘escape’ the molecular basis of the suppression, the other is to grow at a faster rate to offset the inhibitor’s suppression. GMNR disallowed escape, so the evolutionary outcome was a higher growth rate and increased virulence in the absence of the inhibitor (our Fig. 3, bottom right). In contrast, escape does not imply a change in the virulence optimum because the escape virus merely acts as if immunity does not exist.

The overwhelming evidence on viral and bacterial evolution in response to drugs is that resistance is usually through escape and other mechanisms that do not involve increases in growth rate. Molecular studies of bacteria reveal that antibiotic resistance mutations lie in biochemical pathways by which the drug acts, by blocking drug entry, by detoxifying the drug and even by *reduced* growth rate [41]. When resistance is measured in culture, there is less effect of the drug; if the drug retained efficacy but the microbe evolved to grow faster, the drug would still have the same quantitative effect, just in a different dynamic range. Anti-viral drugs are typically overcome by evolution in the target gene [42–47]. Drugs operating by different mechanisms are used so that resistance to one drug does not confer resistance to another; were the basis of resistance faster growth, growth-rate resistance should operate against all inhibitors. Detailed molecular studies often reveal that resistance operates through reduced binding of the drug. Generalized resistance mechanisms are sometimes observed, such as increased mutation rate [45], but increased growth rate has not been reported. Indeed, bacterial persisters comprise a form of generalized resistance to antibiotics that is one of essentially no growth [48].

Likewise, for viruses that evolve in response to immunity, the mechanism best documented is escape (albeit that escape may be the most easily documented mechanism). Influenza is the classic example that virus evolving in its hemagglutinin gene to evade immunity [49]. Norovirus is a second example of evolution to escape immunity [50–52]. One of the benign human coronaviruses also shows this [53].

An exception to the apparent generality of an escape response was reported for a rodent malarial parasite treated with sub-inhibitory concentrations of a drug. The more-virulent line grew faster than the less-virulent line and transmitted better [54, 55], consistent with the GMNR model. These experiments were short term and did not provide a prolonged opportunity for escape mutations to arise, but they nonetheless point to the feasibility of a growth response.

## REVISITING THE MDV EVOLUTION

Evidence is compelling that the early MDV vaccines have at least maintained the hypervirulent strains of MDV, if not been responsible for their initial evolution [1]. Thus, the most highly-virulent strains of MDV transmit more from vaccinated birds (and from the unvaccinated young of vaccinated mothers) than from unvaccinated young of unvaccinated mothers because the latter die so fast—MDV has adapted to the vaccine at the cost of the well-being of the unvaccinated. In the absence of vaccination, MDV transmission is limited by very rapid mortality (their Fig. 1). The GMNR model seems to apply, as the early Marek’s vaccines did not block infection or transmission (a case of their r4 intervention); the data are too limited to infer that the highly virulent strains kill faster because of a high viral growth rate, but that issue is secondary to the high mortality.

The details of this evolution are unclear. It could be that MDV gradually evolved higher virulence over prolonged use of imperfect vaccines. Alternatively, wild-type MDV may sporadically spawn high-virulence mutants that died out in the pre-vaccine era but ascended in populations of imperfectly vaccinated birds. Read *et al*. [1] are specifically agnostic to whether the vaccines explain the origin of hypervirulent strains but suggest that the vaccines were at least responsible for the maintenance of them.

Despite our detailed re-evaluation of the GMNR model, we emphasize that we have no explanation for why MDV evolved to kill the unvaccinated when other viruses (e.g. human viruses) have not, nor have we found a compelling explanation in the literature. We can give reasons that the GMNR model does not apply to many or most human interventions, but it is far more desirable to know specifically when GMNR is expected to apply and lead to pernicious evolution. We offer a few observations that may serve as a starting point for understanding this problem, explaining why MDV is unusual. We note that a key difference between MDV and many human vaccines is the very point of GMNR and Read *et al.* [1]—that the vaccine does not fully protect against infection and transmission. But as there are some human vaccines that allow re-infection, we need to explain why those do not lead to the MDV-like outcome. Thus MDV may be special:

1. The agricultural setting of chicken husbandry is ripe for the evolution of a virus with rapid infection. Birds are crowded with a short harvest time. In this environment, a high mortality rate has little cost to the virus, provided the virus achieves a high transmission rate while the host is alive [56–58].
2. MDV transmission in the chicken house does not require contact from a live bird, only that the infected bird shed virus onto the floor. This feature relaxes selection against killing the birds, provided infected birds produce infectious virus before they die [1, 56–58].
3. Vaccination of housed chickens is near 100% for many houses, so selection is more intense than if coverage was moderate (as is true for some human vaccines).
4. MDV is an alpha herpesvirus, a class of virus that typically establishes latent infections with lifetime transmission. MDV is specifically an oncogenic virus, causing tumor formation in its wild-type state [59]. Herpesviruses have many defenses against host immunity, which in turn enable the virus to persist within the host, and superinfection is known for many. This plethora of anti-immune responses may provide the virus with many pathways to evolve immune escape. T-cell responses are important defenses against at least some beta herpesviruses, and the genetic uniformity of chickens in the house may remove the variation in T-cell repertoires important in host population defense against MDV. Genetic uniformity is not expected to be a sufficient explanation for the evolution of hypervirulent MDV, as it should facilitate virulence evolution of other chicken pathogens, but it may be a contributor.

## TOWARD MORE DETAILED MODELS OF IMMUNITY

The time is ripe to integrate evolution of virulence models with advances in our understanding of immunity. In earlier models, the adaptive immune response was treated as the principal factor that restricted the growth of the virus [13] and those models supported the GMNR result in which imperfect vaccines can select higher virulence. Thus, in a naïve host, the wild-type virus grows ahead of the immune response and grows only so fast as to almost reach a lethal density (level at which it causes death) before adaptive immunity suppresses it. In a vaccinated individual, the growth of the wild-type virus is suppressed, and its density never approaches the lethal density. In the absence of escape mutants, viral evolution in a fully vaccinated population returns the virus to close to its former state by selecting a higher viral growth rate, high enough to just offset the effect of prior immunity. Now, the evolved virus in a naïve host grows so fast that it overshoots the host-death threshold.

More recently, this view of immune control has been revised, and it now seems that the initial control of the pathogen, both in primary and secondary responses, is often by some combination of innate immunity and resource limitation [60–62]. Eventually, the infection is cleared by adaptive immunity, but adaptive immunity develops too late to account for the initial control. This alternative view of viral control may have a profound effect on evolution to escape immunity. A critical point about innate immunity is that it targets highly conserved properties of infection (e.g. dsRNA, DNA in the cytoplasm), and thus is not easily evaded by viral evolution—except by viral acquisition of new functions that specifically defend against innate immunity; resource limitation may be even more challenging to evade.

This newer understanding of infection control specifies a three-phase model of infection: in Phase 1, initial infection occurs, virus growth begins and the innate immune response is triggered; in Phase 2, some combination of the innate response and resource limitation control and potentially reduce the virus density; in Phase 3: adaptive immunity expands and clears the infection. In this model, the maximum pathogen load, which determines the virulence and level of host mortality, depends predominantly on the interaction of the pathogen with the innate immune response or with limited resources needed for viral expansion. Prior adaptive immunity can result in more rapid control of the infections with the wild-type virus in vaccinated hosts, and this additional control reduces the peak pathogen load and/or the duration of infection and thus lowers the virulence of the infection.

Evolution of the pathogen in response to vaccination allows it to evade prior adaptive immunity and to restore the dynamics of the pathogen to what was observed for infections of wild-type pathogens in unvaccinated hosts. However, as the virulence of the infection is determined by the interaction of the pathogen with the innate immune response (or resource limitation), pathogen evolution that changes its interaction with adaptive immunity may not result in a substantial change in virulence when in naïve individuals. In the context of the GMNR model, innate immunity limits the evolution of virulence of the pathogen in vaccinated individuals because the virus cannot overcome innate immunity or resource limits, it can only escape adaptive immunity.

We do not suggest that this new model applies to every pathogen. Rather, it may apply to some pathogens and thus offers a reason that MDV-like outcomes do not occur. If a pathogen can be determined to fit a model of resource limitation or control by innate immunity, then imperfect vaccination may not generate an MDV-like outcome.

## COVID-19 VACCINES

The recent and ongoing Covid pandemic has revealed that re-infection is at least moderately common, whether of vaccinated individuals or individuals with a history of natural infection. It is an obvious question whether to expect evolution of a MDV-like outcome either in response to the vaccine or in response to natural immunity. For the following reasons, we suggest that it is premature to have confidence in any predictions:

1. SARS-CoV2 is still possibly adapting to humans as a new host; it cannot be construed as being at even a temporary evolutionary equilibrium. Furthermore, measuring intrinsic virulence is especially challenging when population immunity is changing rapidly.
2. Since natural immunity and vaccine immunity both wane, it will be difficult to attribute any evolution in response to waning immunity as due to a vaccine or to natural immunity.
3. Since most vaccines only immunize against the spike protein, evolution of escape may be easier against vaccine immunity than against natural immunity. It will be a challenge to disentangle the causes of any observed evolutionary response.
4. Any MDV-like outcome rests on mortality limiting transmission; the contribution of mortality to limiting SARS-CoV2 transmission so far seems to be small.

Should any high-virulence evolution of SARS-CoV2 occur, it will likely be extremely difficult to attribute separate causes to the vaccine, natural immunity and ongoing adaptation to a new host.

## CONCLUSIONS

The evolution of MDV to kill unvaccinated chickens at much higher rate than vaccinated chickens is worrisome. GMNR provided a start in predicting such outcomes, but a deep understanding of the process and a foundation for accurate prediction is still far off. Fortunately, there is scant evidence that past interventions against human viruses (or even viral evolution to escape natural immunity) have caused such pernicious evolution, and the simple conclusion from this evidence alone is that an MDV-like outcome should not be the default assumption. The same conclusion may be true of some livestock vaccines [other than MDV, e.g. 63], but we should be aware that, it may not be reported if people are not looking. Although MDV evolution so far seems unique, we in fact argue that GMNR-type analysis has considerable merit, just the time has arrived to push it to deeper mechanistic levels. A case for vigilance against MDV-like outcomes is easily made on the grounds that the technologies used for infectious disease intervention are expanding, with a concomitant potential for harmful evolution. The GMNR model was an important start on a problem that was not appreciated at the time, and like many virulence evolution models, it is useful in alerting us to possible outcomes that have some plausibility. This is an important step in guiding both the development of and the monitoring of interventions. There is likewise a compelling motivation to understand the MDV evolution in detail, from the molecular genetics of its different strain virulences to the interaction between virus and chicken immunity.
